# Evaluation of MDA-MB-468 Cell Culture Media Analysis in Predicting Triple-Negative Breast Cancer Patient Sera Metabolic Profiles

**DOI:** 10.3390/metabo10050173

**Published:** 2020-04-27

**Authors:** Wojciech Wojtowicz, Anna Wróbel, Karolina Pyziak, Radosław Tarkowski, Alicja Balcerzak, Marek Bębenek, Piotr Młynarz

**Affiliations:** 1 Department of Biochemistry, Biotechnology and Molecular Biology, Bioanalytical Laboratory, Wroclaw University of Science and Technology, Wybrzeze Wyspianskiego 27, 50-370 Wroclaw, Poland; wojciech.wojtowicz@pwr.edu.pl; 2 Ryvu Therapeutics S.A.; Bobrzynskiego 14; 30-348 Krakow, Poland; anna.wrobel@ryvu.com (A.W.); karolina.pyziak@ryvu.com (K.P.); 3 Lower-Silesian Oncology Centre, pl. Hirszfelda 12, 53-413 Wroclaw, Poland; tarrad@poczta.onet.pl (R.T.); ko-alicja@wp.pl (A.B.); bebenek.m@dco.com.pl (M.B.)

**Keywords:** metabolomics, TNBC, MDA-MD-468, triple-negative breast cancer, ^1^H NMR spectroscopy

## Abstract

Triple-negative breast cancer (TNBC) is characterized by limited survival, poor prognosis, and high recurrence. Understanding the metabolic adaptations of TNBC could help reveal improved treatment regiments. Here we performed a comprehensive ^1^H NMR metabolic characterization of the MDA-MB-468 cell line, a commonly used model of TNBC, followed by an analysis of serum samples obtained from TNBC patients and healthy controls. MDA-MB-468 cells were cultured, and changes in the metabolic composition of the medium were monitored for 72 h. Based on time courses, metabolites were categorized as being consumed, being produced, or showing a mixed behavior. When comparing TNBC and control samples (HC), and by using multivariate and univariate analyses, we identified nine metabolites with differing profiles). The serum of TNBC patients was characterized by higher levels of glucose, glutamine, citrate, and acetoacetate and by lower levels of lactate, alanine, tyrosine, glutamate, and acetone. A comparative analysis between MDA-MB-468 cell culture media and TNBC patients’ serum identified a potential systemic response to the carcinogenesis-associated processes, highlighting that MDA-MB-468 cells footprint does not reflect metabolic changes observed in studied TNBC serum fingerprint.

## 1. Introduction

Triple-negative breast cancer (TNBC) accounts for 10% to 22% of all diagnosed breast cancer cases, has the worst survival prognosis, and is associated with the most problems during treatment [1]. TNBC cells are characterized by the absence of expressions of three receptors: estrogenic receptor alfa (ER), progesterone receptor (PR), and human epidermal growth factor 2 receptor (HER2) [2]. Patients suffering from TNBC have the shortest five-year survival, which strongly depends on the stage (from 92.7% for stage 0 to 26.1% for stage 4, with an overall survival rate of 78.6%) [3]. TNBC treatment is challenging due to the absence of a response to standard hormonal therapy or therapies targeted at the specific receptors (e.g., HER2) [2,3]. Therefore, it is necessary to determine the molecular bases of the biochemical processes governing tumor growth. The development of omics science has enabled the investigation and tracking of metabolic processes in living systems [4,5,6]. This holistic approach allows researchers to address the overall processes occurring in living systems, where ongoing pathological processes are overlap with other factors associated with normal functions. 

Cell cultures are a commonly used model of cancer metabolism. Culture methods allow for the study of the external and internal responses of cancer cells in isolation, which permits a high level of control over nutrient availability [7,8]. Cell culture analysis has become one of the most important methods in biological sciences, revealing much about cellular processes [9,10,11]. It is also believed that 3D cell cultures can be used as an external model of tumors. Furthermore, culture methods are widely used for the monitoring of ongoing internal (fingerprinting) and external (footprinting) metabolic processes [12,13,14,15,16]. Moreover, in some cases, the metabolomics of the cells’ footprint might be treated as a link between in vivo (human serum) and in vitro information [16]. However, today, monolayer cell culture is often considered a limited model of metabolism [17,18]. Among TNBC cases, various cell lines are available and correspond to various subtypes [19]. These TNBC model systems were metabolically investigated in a variety of studies to the search for metabolic vulnerabilities connected to EGFR, MET [20] or MUC1 [21], induced methionine stress [22], and anticancer drug response [23].

The undertaken study was performed to check on whether an in vitro isolated model and an in vivo complex system could be easily linked, based on major metabolites changes. We tested the extent to which the metabolic footprint of MDA-MB-468 cells with a high Ki-67 level [19] share common features with the metabolic profile obtained from the intact serum of TNBC patients. To test this, using a high sampling rate, we monitored the metabolic composition of cell media to obtain the patterns of uptake and secretion of metabolites over 72 h in culture and then compared these data to changes in human serum metabolites.

## 2. Results

The cell line media ^1^H NMR spectra analysis identified 29 metabolites in one series of experiments. The cell lines cultured in the Dulbecco’s Modified Eagle medium (DMEM) showed a proliferation at the level of 174,000–440,000 cells. Each time point was measured in triplicate. During the tracking of metabolite levels in cell media, three trends were distinguished: increasing (corresponding to secretion), decreasing (corresponding to uptake), and mixed (corresponding to a metabolic switch during the time of the experiment). The multivariate analysis performed for all triplicates and time intervals based on PLS-DA models showed continuous trend of changes and stability during cell culturing (Figure S1). 

The NMR spectra of blood serum samples allowed us to determine 31 metabolites, four unknown signals, and nine ranges of chemical shift regions assigned to different lipid types (Supplementary Table S2). Demographic information about patients enrolled in studies is presented in Table 1. Detailed medical information about TNBC patients are presented in Table S1. Matching metabolites in both studies are presented in Supplementary Table S3.

**Figure 1:**
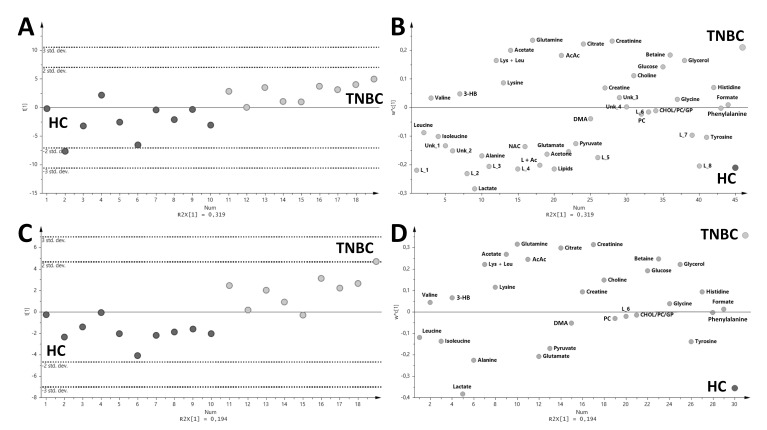
The PLS-DA score (**A**,**C**) and loadings (**B**,**D**) plots for triple-negative breast cancer patients (TNBC, light gray) and healthy control (HC, gray). (**A**,**B**) PLS-DA models with all variables used in the analysis (R2X = 0.194, R2Y = 0.69 and Q2 = 0.438, CV-ANOVA, *p* < 0.05). (**C**,**D**) PLS-DA models without the selected variables (Table S2) (R2X = 0.319, R2Y = 0.547 and Q2 = 0.377, CV-ANOVA, *p* < 0.05).

The low occurrence of TNBC among breast cancer patients led to the need for balanced study groups to perform multivariate data analysis (MVA). The performed PLS-DA analysis based on the serum ^1^H NMR relative integral data was carried out with the use of selected samples by the Kennard-Stone algorithm (Figure 1). 

The HC vs. TNBC PLS-DA analysis showed intergroup separation within the model parameters R2X = 0.194, R2Y = 0.69, and Q2 = 0.438, CV-ANOVA, *p* < 0.05 (Figure 1A,B), validated by the CV-ANOVA method and R2X = 0.319, R2Y = 0.547, and Q2 = 0.377, CV-ANOVA, *p* < 0.05 (Figure 1C,D). The multivariate analysis showed mainly the change in the levels of glutamine, citrate, creatinine, acetate, acetoacetate, betaine, glucose, glycerol, choline, leucine, and lysine, which were up-regulated in TNBC. Meanwhile, all lipid levels were down-regulated together with lactate, alanine, acetone, glutamate, pyruvate, tyrosine, and isoleucine in TNBC relative to HC. Univariate statistical analysis revealed only nine metabolites that were changed in the HC vs. TNBC comparison (Table 2). Additionally, seven signals assigned to the lipid fraction were found to be statistically significant (Table 3). Glucose, glutamine, citrate, and acetoacetate were up-regulated in the TNBC group relative to the HC group, while lactate, alanine, tyrosine, glutamate, and acetone were down-regulated (Table 2). Seven lipids were significantly down-regulated in the TNBC group (Table 3, Altogether, the levels of 16 metabolites were significantly different when comparing the TNBC and HC groups.

**Figure 2:**
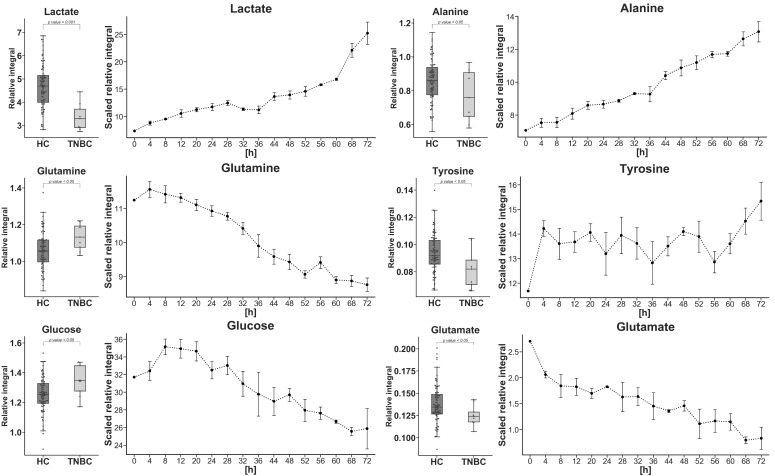
Statistically important metabolites identified from the HC vs. TNBC (box plots) comparison with matching scaled relative integral from MDA-MB-468 medium extracts. Data points in the line plots were calculated based on the mean values of triplicates for each time interval with standard deviation error bars. In the boxplot: whiskers = 1.5 × IQR, lines = average; boxes = interquartile range Q1–Q3.

Next, statistically important metabolites identified in the serum of TNBC patients were compared to the cell culture footprint results (Figure 2). When comparing the lactate, alanine, glutamine, tyrosine, and glucose profiles between the cell media and blood serum of patients suffering from TNBC, we detected opposite trends (Figure 2).

**Figure 3:**
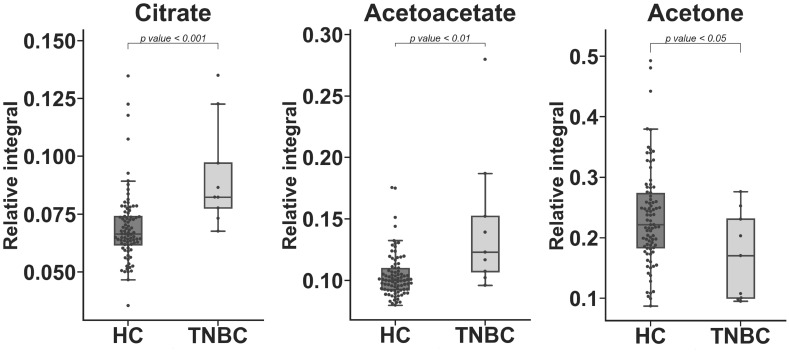
Statistically important metabolites identified in the HC vs. TNBC comparison but not identified in the cell line medium experiment. Whiskers = 1.5 × IQR; lines = average; boxes = interquartile range Q1–Q3.

**Figure 4:**
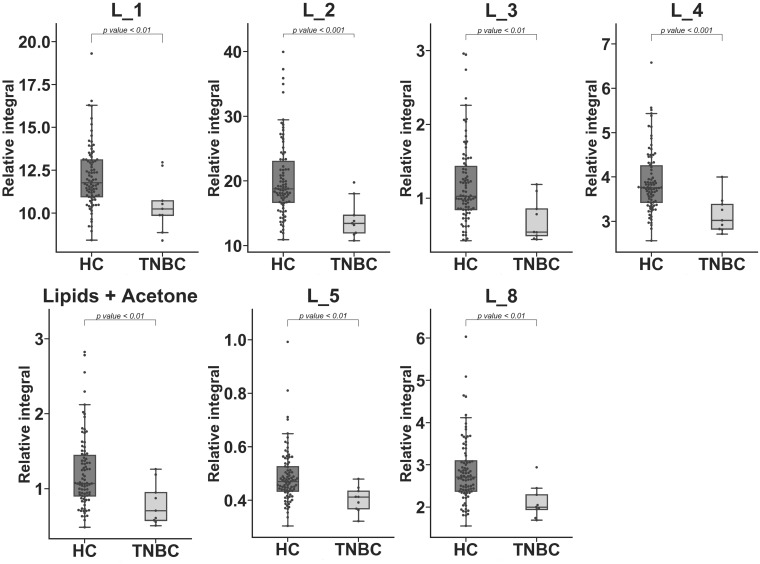
Statistically important lipid fragments identified in the HC vs. TNBC comparison but not identified in the cell line medium experiment. Whiskers = 1.5 × IQR; lines = average; boxes = interquartile range Q1–Q3.

Three significantly different metabolites (HC vs. TNBC) were identified only in serum samples and could not be detected in media samples: citrate, acetoacetate, and acetone (Figure 3). In the case of lipid classes, seven were statistically important in serum (Table 3, Figure 4). The chemical shift assignment for these fragments is presented in Supplementary Table S2. Lipid fragments could not be assigned in cell media samples due to the method of sample preparation.

**Figure 5:**
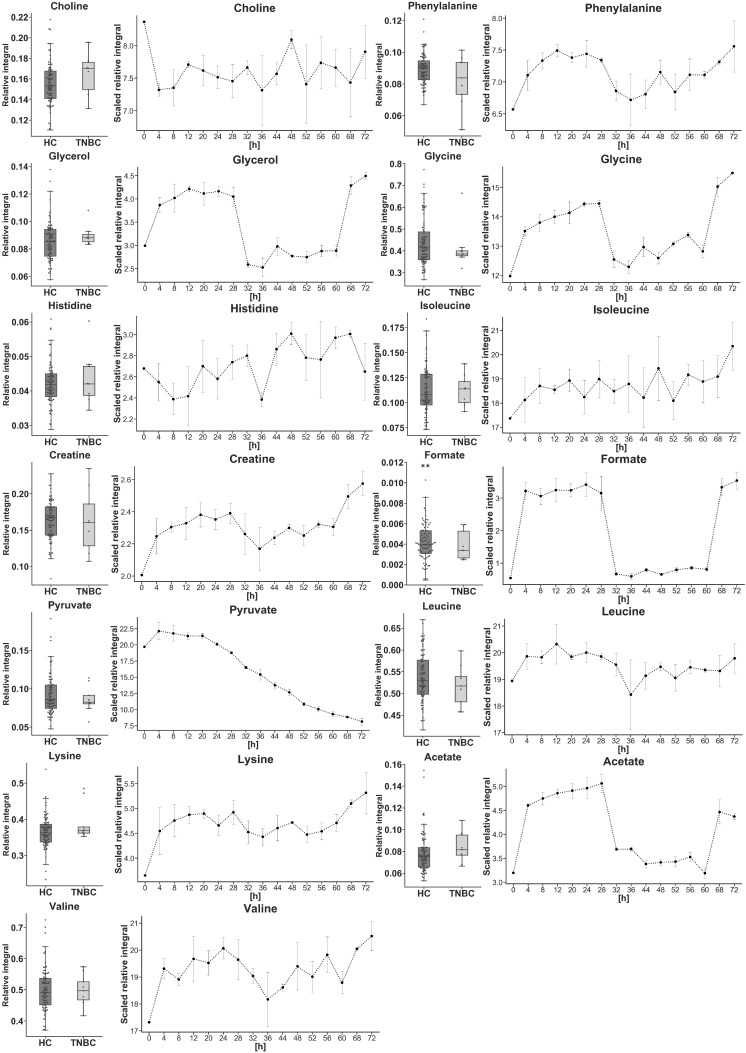
Identified non-statistically important metabolites HC vs. TNBC in serum (box plots) with the matching metabolites’ resonance signals from the cell culture experiments. The data points in line plots are the mean values of triplicates for each time interval with standard deviation error bars . In the boxplots: whiskers = 1.5 × IQR; lines = average; boxes = interquartile range Q1-Q3; ** extreme outlying observation beyond the plot range.

The remaining identified and matching metabolites (between time-dependent cell cultures and serum blood samples HC vs. TNBC, which were not statistically significant) are shown in Figure 5. The time courses of these metabolites included three types of changes—increasing, decreasing, and mixed. 

**Figure 6:**
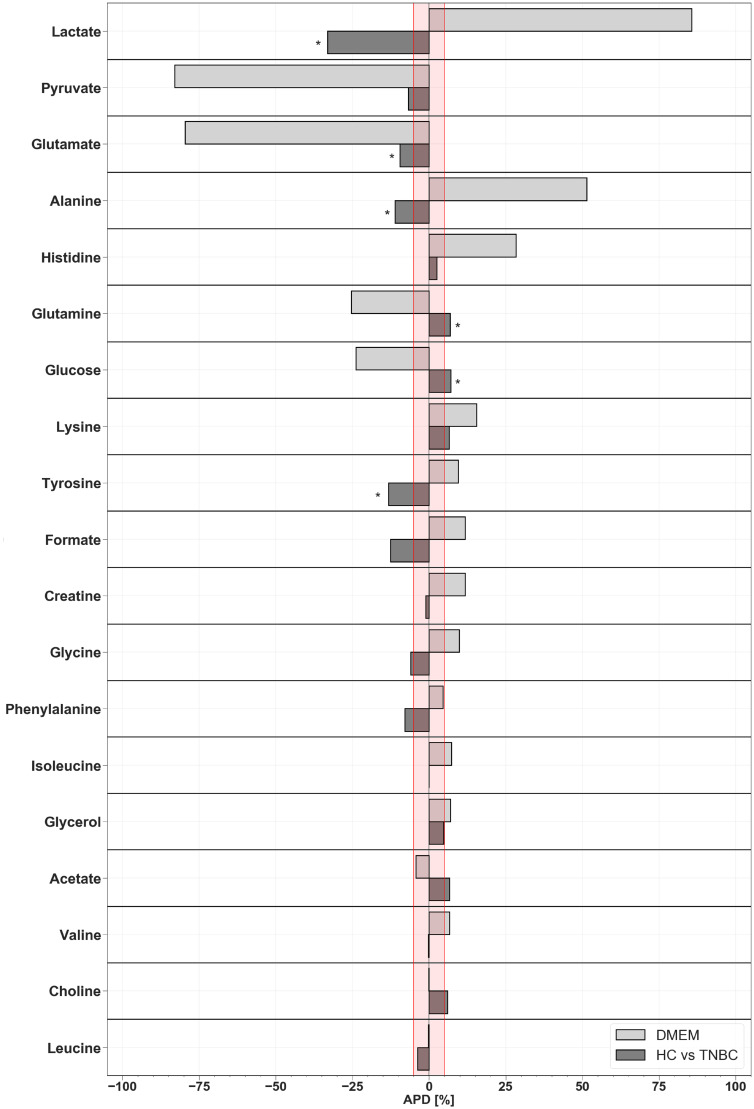
Comparison of the average percentage differences (APD) of matching metabolites between cell cultures and patient’s serum samples (TNBC vs. HC). *Statistically significant metabolites. The red line indicates the <−5, 5> range.

The comparison highlighting trends in changes of metabolites in the blood serum of TNBC patients and cell media is given in Figure 6.

## 3. Discussion

Cell lines are a common model choice for tumor metabolism studies, and MDA-MB-468 is a widely used model for TNBC [20,21,22,23]. This experiment provides information about the influx and outflow of metabolites for which, depending on the time of cell cultivation, different results could be obtained. Especially when tracking the metabolites whose levels were related to the cell developmental stage. The medium contents and metabolites that were assigned are given in Supplementary Table S4.

Although a different method of sample preparation (methanol precipitation) was used in the cell culture experiments than in the serum samples (intact), to avoid variability resulting from the sample preparation methods, comparisons were based on the average percentage differences (APD) calculated from two separate datasets. Moreover, each set of data (serum, DMEM) was separately normalized and scaled to overcome this limitation. 

A comparison of the identified metabolites between in vivo and in vitro experiments, revealed that 19 serum metabolites coincided with culture medium metabolites. However, 21 were unique for serum samples. When comparing the statistically significant data obtained for the patient’s biological material (HC vs. TNBC comparison) (Table 2) and metabolites in the composition of media extracts, it is necessary to consider only metabolites that are overlap in two studied cases. These metabolites include lactic acid, alanine, glutamine, tyrosine, glucose, and glutamic acid (Figure 1). The direct comparison of all overlying metabolites between in vitro and in vivo experiments showed the differences in organism and in vitro response. The opposite trend was exhibited by serum lactate and medium lactate, which in cell culture is an excreted byproduct and increasing, while its value in blood serum of TNBC patients was lower than in HC. Lactate can be used as a source of energy but it also lowers the pH in the tumor microenvironment and has an immunosuppressive effect [24], which can explain its increase in the cell medium. Another explanation could be the reverse Warburg effect, in which the lactate and pyruvate are used as highly energetic fuel to drive cancer cell proliferation, thereby contributing to poor prognosis among TNBC patients [25,26]. The general trend of lactate in different studies of breast cancer (BC) in vivo samples is up-regulation [27,28]; these results are in contradiction to this study for TNBC serum samples.

In this study, among two ketone bodies (acetoacetate, acetone) found in serum only, the level of acetone decreased. This metabolite is regarded as a product of a non-enzymatic (spontaneous) process of acetoacetate breakdown [29]. In addition, among statistically significant changes in the patients’ serum, it is evident that the acetoacetate level is an important differentiating compound and is significantly elevated in the TNBC group (Table 2). Acetoacetate was found only in sera and not in media. Acetoacetate is a ketone body that can be produced and released by the liver, particularly under conditions of high rates of fatty acid beta-oxidation. The higher level of acetoacetate in TNBC patients in the studied biofluid is consistent with the literature [30]. The recent studies performed with 3-hydroxybutyrates and acetoacetate as a medium content did not interfere with the proliferation of studied cell lines [31]. These metabolites might be associated with an organism’s response to the ongoing disease.

Alanine and tyrosine also decreased in the TNBC serum samples group. For the medium extracts, the levels of these metabolites increased over time. Alanine can be reversibly transformed by alanine transaminase to pyruvate and used as an energy source. The decreasing trend for in vivo samples was detected for alanine, which is consistent with the findings of Shen et al. [32]. In the case of tyrosine, its use is likely related to transformation in the biochemical pathway to fumaric acid and acetoacetate [33]. The tyrosine level changes detected in the serum are also in agreement with the literature [32,34,35,36].

Glucose and glutamine levels increased in the serum of TNBC patients. These amino acids play significant roles in the development of tumor cells, and TNBC is considered glutamine and glucose-dependent [37,38,39,40]. Interestingly, the relative integral of these compounds (glucose and glutamine) and their elevated level in the serum of TNBC patients (vs. the HC group) (Table 2, Figure 3) is the reverse of the finding for the cell culture experiments. These in vivo results are likely associated with simultaneously decreased lactate, which can be transformed into glucose in the Cori cycle [41]. In case of glutamine from in vivo samples, the situation is unclear, with some reports showing an up-regulation in a BC relative to an HC group [42], while other studies reported no change [32] or a decrease in the BC group [36,42]. However, to our knowledge, only Shen et al. have investigated the TNBC subgroup, the remaining studies focused primarily on a general approach to BC. 

The relative integral of citrate is increased in serum TNBC samples. However, in the medium extracts, the resonance signal was not quantified. The serum citrate increase can be associated with a site-specific bottleneck point in the tricarboxylic acid cycle (TCA) or/and associated with the organism’s systemic response as an antitumor function agent [43]. Moreover, previously, the increase in serum citrate was observed between early breast cancer (EBC) and metastatic breast cancer (MBC) [44]. 

Most metabolites identified as statistically important in serum samples expressed the opposite trend in medium extracts (Figure 6). 

In the cell line model, the biochemical machinery is strictly isolated, removing the complication of whole-organism biochemical pathways, which might produce very different final results. Also, the technical parameters that characterize in vitro experiments (e.g., a short experiment timeline and the availability of medium nutrients, monolayer or 3D cell culturing, sample preparation, used analytical methods) might contribute to the differences we detected when comparing these two biological systems (cell medium vs. serum). Moreover, the literature results reveal that depending on the TNBC cell line, it can exhibit different behavior, influencing the final outcome [45,46,47]. These studies show the limitation of experimenting on a single cell line where an observable overview is narrowed; however, it offers a precise examination of the MDA-MB-468 cell line.

Another difficulty encountered in this analysis is metabolites binding to a protein in intact serum samples (as reported in the literature), this problem does not occur in methanol precipitated samples [48]. This might contribute to the differences in relative concentration between the studied materials. However, the changes identified within a specific experiment dataset (represented by APD) should reflect variation among identified metabolites in the studied biological material. With the ability to determine metabolites that were important in the in vivo and in vitro experiment, the interpretation of possible changes and differences in metabolome could be helpful when designing future experiments. 

Finally, the interpretation of the results with literature data must be carried out with great caution. The alterations in the metabolite sets among the studied groups might vary depending on the experiment design, the number of samples, and ethnic homogeneity (MDA-MB-468, African American origin; Serum, Caucasian) [49] or reference group type [27,34]. 

## 4. Materials and Methods

### 4.1. Sample Collection

Peripheral venous blood samples were drawn from all of the participants after overnight fasting (Approval no. 4/BO/2015). Blood samples were collected using serum tubes (Sarstedt S Monovette system—silicate activator system, Sarstedt AG & Co., Rheinbach, Germany) and then centrifuged at 1000 RCF for 15 min at 4 °C. The serum samples were stored in 1.5-ml Eppendorf tubes and kept at −80 °C until analysis.

The data calculations were carried out on nine serum samples of TNBC patients with an established diagnosis and 86 control samples, which were part of a larger in-house serum database of breast cancer and control subjects. All of the subjects were patients of the Lower Silesian Oncology Center. The serum samples were taken before any treatment had started. The medical and demographic information of enrolled subjects is presented in Table 1 and Supplementary Table S1.

### 4.2. Cell Culturing in DMEM

Before the experiment, the cells were cultivated in DMEM Low-Glucose (5.5 mM) culture medium (Glutamine 3.24 mM, with bicarbonate) (Supplementary Table S4) (Sigma Life Science, Sigma-Aldrich, Gillingham, UK) with 10% fetal bovine serum (FBS) (Biowest, origin South America, Riverside, MO, USA) and penicillin-streptomycin (HyClone, GE Healthcare Life Sciences, Wien, Austria). The MDA-MB-468 cells were seeded in six-well plates at a density of 173,000 cells per well. For each well, 4 mL of DMEM, Low-Glucose culture medium (Sigma Life Science, Sigma-Aldrich, Great Britain) with 10% FBS (Biowest, origin South America, Riverside, MO, USA) and penicillin-streptomycin (HyClone, GE Healthcare Life Sciences, Wien, Austria) were added. The cell culture was maintained in a CO_2_ incubator (Binder, Tuttlingen, Germany) under 5% CO_2_ conditions at 37 °C and 80% humidity. Each time interval was prepared in triplicate and in separate wells. The final cell density at 72 h was 440,000 cells per well. The culture medium was not exchanged during the experiment. The medium was collected in 4- and 8-h time intervals.

### 4.3. Medium Sample Preparation for NMR Measurements

At each time point of the experiment, 1 mL of cell culturing medium was collected from each triplicate. The collected medium had been frozen and stored at −80 °C. Before analysis, medium samples were thawed at room temperature and vortexed. From each sample, 400 μL of medium were transferred to a new Eppendorf tube with 1.2 mL of methanol (LC-MS grade, Merck, Darmstadt, Germany). The mixture of medium-methanol was shaken for 10 min at 30 Hz (Tissiulyzer LT, Qiagen, Germantown, MD, USA) and incubated at −20 °C for 20 min. Shaking was repeated for 5 min at 30 Hz. The mixture was then centrifuged for 30 min at 4 °C, 21,885 RCF. The supernatant (1 mL) was transferred to a new Eppendorf tube and evaporated to dryness under vacuum centrifuge (JWElectronic WP-03, Warsaw, Poland) at 40 °C, 221 RCF. The evaporated medium extracts were resuspended in 600 μL PBS buffer (pH, 7.4, 20% D_2_O, 0.01 µM TSP), then vortexed. Finally, 550 μL was transferred to an NMR cuvette (5 mm, SP type, ARMAR Chemicals, Döttingen, Switzerland). Prepared samples were stored at 4 °C until NMR spectra measurement.

### 4.4. Serum Sample Preparation for NMR Measurements

The collected serum samples were thawed at room temperature and vortexed. From each serum sample, 200 μL was mixed with 400 μL saline solution (0.9% NaCl, *w/v*) containing 20% D_2_O. The mix of serum and saline solution was vortexed then centrifuged for 10 min at 4 °C, 14,006 RCF. Overall, 550 μL of supernatant from each sample was transferred into a 5-mm NMR tube (SP, 5 mm, ARMAR Chemicals, Döttingen, Switzerland). Samples were kept at 4 °C before measurement.

### 4.5. NMR Measurements

The NMR spectra of serum and cell media samples were recorded at 300 K using the Avance II spectrometer (Bruker, GmBH, Bremen, Germany) operating at a proton frequency of 600.58 MHz. The 1D ^1^H NMR spectra were recorded using a *cpmg1dpr* pulse sequence with water presaturation (Bruker notation city). For each sample, 128 scans were collected with spin-echo delay of 400 μs; 80 loops; relaxation delay of 3.5 s; acquisition time of 2.73 s; time domain of 64k; and spectra width of 20.01 ppm. 

### 4.6. Metabolites Identification NMR

The metabolite resonance signals were identified in accordance with assignments published in the literature, available in the Chenomx software (v 8.2 Chenomx Inc., Edmonton, AB, Canada) and online databases Biological Magnetic Resonance Data Bank [50] and Human Metabolome Database [51].

### 4.7. Processing for Data Analysis

The medium and serum samples were calculated in different data matrixes. The 1D ^1^H NMR spectra were processed with a line broadening of 0.3 Hz, manually phased, baseline-corrected with MestReNova software (Mestrelab Research v 11.0), and referenced to glucose anomeric carbon signal group δ = 5.225 ppm for serum samples and to TSP signal δ = 0.000 ppm for medium samples. The spectra range for water resonance signals was removed from the data matrix. The alignment of resonance signals was done using the correlation optimized warping algorithm (COW) [52] and the icoshift algorithm implemented in MATLAB (v R2014a, MathWorks Inc., Natick, MA, USA) [53]. Spectra from serum were normalized with PQN (Probabilistic Quotient Normalization) in a separate data matrix [54]. The cell media experiment data matrix was normalized to the TSP resonance signal. The relative integral of NMR measured metabolite was obtained as a sum of data points of the non-overlapping resonances or a cluster of partly overlapping resonances. All data analyses were carried out in MATLAB software (MATLAB v. R2014a, MathWorks, Inc. Natick, MA, USA).

### 4.8. Univariate Data Analysis

The HC vs. TNBC statistics were calculated on representative data sets containing nine samples for TNBC and 86 samples for HC. The relative integral of assigned metabolites was used for calculations for univariate statistics. The Shapiro-Wilk test was calculated for normality verification for each variable. The equality of variances for normally distributed data was tested using an F-test. Depending on the results of the normality and variance tests, a parametric (equal/unequal variance student’s *t*-test) or nonparametric (Mann-Whitney-Wilcoxon test) test was used. All statistical tests were calculated at the significance level of α = 0.05. 

The changes between the metabolite relative integrals of studied groups were verified by the average percentage difference (APD). Average percentage differences for cell culture experiments were calculated between the mean of the first three (0–4–8-h) time interval and the mean of the last three (60–68–72-h) time points. The data points in line plots were calculated based on mean values of triplicates for each time intervals.

### 4.9. Multivariate Data Analysis

The MVA analysis was carried out on relative metabolite integrals. All the relative integral variables were scaled to unit variance (UV). The Principal Component Analysis (PCA) was applied to achieve data overview and outlier detection. The MVA data visualization has a marked ellipse with Hotelling’s T2 range (95%). The PLS-DA models’ reliability was assessed by cross-validation analysis of variance (CV-ANOVA) at a significance level of α = 0.05. MVA calculations were done using SIMCA 14.1 (Sartorius Stedim Biotech, 2017).

Patients were age- and menopause status-matched. Samples for MVA calculation were selected based on the Kennard-Stone algorithm. The PLS-DA models for serum samples were calculated on two different variable sets first, containing all variables in the relative integral data matrix; and second, without the most intense ^1^H NMR lipid fragments and specified resonance signals (excluded metabolites according to Supplementary Table S2: 1, 5, 6, 8, 11, 15, 16, 18, 19, 20, 26, 29, 30, 39, and 40).

## 5. Conclusions

Comparison of the ^1^H NMR-based extracellular metabolome profiles of in vitro cultured MDA-MB 468 cells with those of TNBC patient serum blood samples allowed for the parsing out of the important metabolic differences between these two biomaterials. Study results imply that metabolites secreted by studied cancer cells into the surrounding microenvironment are not noticeable in the same manner as they are in patient serum. Moreover, exactly these metabolites in a TNBC patient’s serum composition are in significant deficit in comparison to the control group (lactate, alanine). The detected differences in the metabolite profiles are likely related to changes arising from the response to the disease. Most observed statistically important metabolites for TNBC patient’s serum were at opposite levels compared to the measured compounds in cell medium (expect glutamate) which show that studied cell line footprint do not replicate enrolled patients’ serum fingerprints. These findings could lead to better verification of whole-organism biochemical response to TNBC cancer, as well as identification of the possible differences between in vivo (organism) and in vitro (local) metabolism, thereby highlighting the limitations of cell culture models.

## Figures and Tables

**Table 1:** Medical and demographic information about patients enrolled in the study.

Group	Average Age	Range	*p* Value		
TNBC (9)	56.67	50–67	0.79		
HC (86)	57.41	45–68			
Menopausal status	Pre-menopausal	Post-menopausal	N.D		
TNBC	0	9	0		
HC	0	86	0		
Comorbidity	Diabetes	Hypertension	Hypothyroidism	Hyperthyroidism	N.D
TNBC	0	4	1	1	0
HC	4	34	14	0	2
Smoking	Smokers	Non-smokers	N.D.		
TNBC	-	-	-		
HC	27	55	4		

N.D.—No data.

**Table 2:** Metabolites with significantly different profiles when comparing HC vs. TNBC, sorted by *p*-value.

Metabolite	TNBC vs. HC APD [%]	Coefficient of Variation		*p* Value
		HC	TNBC	
Lactate	−33.149	19.889	17.203	6.26 × 10^−5 a^
Citrate	27.819	22.291	24.960	5.10 × 10^−4^ ^c^
Acetoacetate	32.928	17.322	40.115	2.44 × 10^−3 c^
Tyrosine	−13.204	14.847	17.841	1.98 × 10^−2 b^
Glucose	7.068	9.307	7.745	2.58 × 10^−2 a^
Glutamine	6.860	9.315	6.373	2.87 × 10^−2 a^
Glutamate	−9.490	15.409	9.123	3.89 × 10^−2 c^
Acetone	−31.267	34.257	42.560	4.01 × 10^−2 c^
Alanine	−11.149	14.519	19.592	4.45 × 10^−2 a^

^a^*t*-test for equal variances, ^b^*t*-test unequal variances, ^c^ Mann-Whitney-Wilcoxon test.

**Table 3:** Percentage change between statistical significance assigned signals originating from the lipid fraction in the HC and TNBC serum, sorted by *p*-value.

Metabolite	TNBC vs. HC APD [%]	Coefficient of Variation		*p* Value
		HC	TNBC	
L_2	−35.525	29.061	20.954	6.45 × 10^−4 c^
L_4	−20.872	17.941	13.087	9.75 × 10^−4 c^
L_8	−29.523	27.006	18.362	1.81 × 10^−3 c^
L_5	−18.917	20.758	11.910	2.24 × 10^−3 c^
L_3	−50.287	45.304	39.963	3.40 × 10^−3 c^
Lipid + Acetone	−41.161	39.546	35.046	4.69 × 10^−3 c^
L_1	−14.663	15.130	14.784	6.41 × 10^−3 c^

^c^ Mann-Whitney-Wilcoxon test.

## Supplementary Materials

The following are available online at https://www.mdpi.com/2218-1989/10/5/173/s1: Figure S1: The PLS-DA models for all triplicates for time intervals in cell medium experiment based on the identified metabolites relative integral, Table S1: Medical information of triple negative breast cancer patients enrolled in the study, Table S2: Metabolites assignments for serum samples with observed chemical shift of signals used for calculations of relative integral along with HMDB ID, Table S3: Metabolites assignments for matching metabolites in cell culture with observed chemical shift of signals used for calculations of relative integral along with HMDB ID, Table S4: Cell media composition provided by the manufacturer.
