# Supplementary material for: Evaluation of MDA-MB-468 Cell Culture Media Analysis in Predicting Triple-Negative Breast Cancer Patient Sera Metabolic Profiles
Source: Metabolites. 2020 Apr 27;10(5):173. doi: 10.3390/metabo10050173 (PMC7281562; doi:10.3390/metabo10050173)
Supplement: Supplementary file 1 [file metabolites-10-00173-s001.pdf]

SUPPLEMENTARY DATA

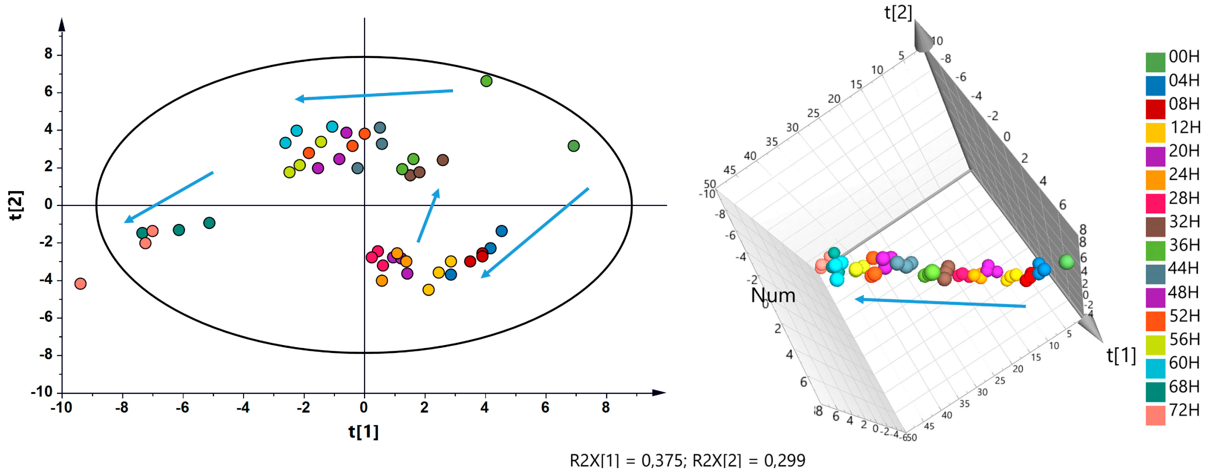

Figure S1. The PLS-DA models for all triplicates for time intervals in cell medium experiment based on the identified metabolites relative integral.

Table S1. Medical information of triple negative breast cancer patients enrolled in the study.

Triple negative breast cancer patients group				
Stage	I	II	III	N.D.
N=	4	2	1	2
HER2 status	Her+	Borderline	Her-	N.D.
N=	0	2	6	1
ER status	ER+	ER-	N.D.	
N=	0	9	0	
PgR status	PgR+	PgR-	N.D.	
N=	0	9	0	
Ki-67 level*	Low	Intermediate	High	N.D.
N=	3	0	5	1

* Ki-67 expression classification: High ≥ 20%, Intermediate 19% - 14% and low <14%.
Her2(+/borderline/-) - human epidermal growth factor receptor 2 (positive/borderline/negative). ER(+/-) – estrogen receptor (positive/negative), PgR(+/-) – progesterone receptor (positive/negative), Ki-67 - cellular marker for proliferation.

Table S2. Metabolites assignments for serum samples with observed chemical shift of signals used for calculations of relative integral along with HMDB ID.

No.	Signal assignment	Chemical shift	HMDB ID
1	L_1 (LDL/VLDL)*#	0.85 ppm	-

2	Leucine	0.96 ppm	HMDB0000687
3	Valine	0.99 ppm	HMDB0000883
4	Isoleucine	1.02 ppm	HMDB0000172
5	Unk_1 (Doublet) #	1.15 ppm	-
6	Unk_2 (Singlet) #	1.18 ppm	-
7	3-Hydroxybutyrate	1.20 ppm	HMDB0000357
8	L_2 (LDL/VLDL)* #	1.28 ppm	-
9	Lactate	1.33 ppm	HMDB0000190
10	Alanine	1.48 ppm	HMDB0000161
11	L_3 (Adipic acid) #	1.58 ppm	-
12	Lys + Leu (Lysine + Leucine)	1.71 ppm	-
13	Lysine	1.89 ppm	HMDB0000182
14	Acetate	1.92 ppm	HMDB0000042
15	L_4 (CH2C=C) #	2.01 ppm	-
16	NAC (N-acetylated compounds) #	2.04 ppm	-
17	Glutamine	2.12 ppm	HMDB0000641
18	Lipids + Acetone# (L + Ac)	2.21 ppm	-
19	Acetone#	2.23 ppm	HMDB0001659
20	Lipids#	2.25 ppm	-
21	Acetoacetate (AcAc)	2.28 ppm	HMDB0000060
22	Glutamate	2.34 ppm	HMDB0003339
23	Pyruvate	2.37 ppm	HMDB0000243
24	Citrate	2.53 ppm	HMDB0000094
25	Dimethylamine	2.72 ppm	HMDB0000087
26	L_5 (C=CCH2C=C) #	2.75 ppm	-
27	Creatine	3.04 ppm	HMDB0000064
28	Creatinine	3.05 ppm	HMDB0000562
29	Unk_3 (Singlet) #	3.14 ppm	-
30	Unk_4 (Singlet) #	3.15 ppm	-
31	Choline	3.2 ppm	HMDB0000097
32	O-Phosphocholine (PC)	3.21 ppm	HMDB0001565
33	L_6	3.22 ppm	-
34	Chol/PC/GPC/LIPID	3.17 ppm	-
35	Glucose	3.25 ppm	HMDB0000122
36	Betaine	3.27 ppm	HMDB0000043
37	Glycine	3.56 ppm	HMDB0000123
38	Glycerol	3.58 ppm	HMDB0000131
39	L_7 (glycerol of lipids) #	5.19 ppm	-
40	L_8 (poly-UFA/fatty acyl groups) #	5.30 ppm	-
41	Tyrosine	6.90 ppm	HMDB0000158
42	Histidine	7.04 ppm	HMDB0000177
43	Phenylalanine	7.32 ppm	HMDB0000159
44	Formate	8.46 ppm	HMDB0000142

* LDL-low-density lipid; VLDL- very low-density lipid, # Resonance signals removed in second PLS-DA model. Poly-UFA - polyunsaturated fatty acids.

Table S3. Metabolites assignments for matching metabolites in cell culture with observed chemical shift of signals used for calculations of relative integral along with HMDB ID.

No.	Signal assignment	Chemical shift	HMDB ID
1	Leucine	0.96 ppm	HMDB0000687
2	Isoleucine	1.01 ppm	HMDB0000172
3	Valine	1.04 ppm	HMDB0000883
4	Alanine	1.49 ppm	HMDB0000161
5	Acetate	1.92 ppm	HMDB0000042
6	Glutamate	2.35 ppm	HMDB0003339
7	Pyruvate	2.38 ppm	HMDB0000243
8	Glutamine	2.46 ppm	HMDB0000641
9	Lysine	3.03 ppm	HMDB0000182
10	Creatine	3.04 ppm	HMDB0000064
11	Choline	3.21 ppm	HMDB0000097
12	Glucose	3.25 ppm	HMDB0000122
13	Glycine	3.57 ppm	HMDB0000123
14	Glycerol	3.66 ppm	HMDB0000131
15	Lactate	4.12 ppm	HMDB0000190
16	Tyrosine	6.91 ppm	HMDB0000158
17	Histidine	7.11 ppm	HMDB0000177
18	Phenylalanine	7.43 ppm	HMDB0000159
19	Formate	8.46 ppm	HMDB0000142

Table S4. Cell media composition provided by the manufacturer.

**DMEM medium (D6046)**		**Assigned in ^{1}H NMR spectra**
**Component**	g/L	
**Inorganic Salts**		
$CaCl_2$	0.2	-
$Fe(NO_3)_3 \bullet 9H_2O$	0.0001	-
$MgSO_4$	0.09767	-
KCl	0.4	-
$NaHCO_3$	3.7	-
NaCl	6.4	-
NaH_2PO_4	0.109	-
**Amino Acids**		
L-Arginine • HCl	0.084	-
L-Cystine • 2HCl	0.0626	-
L-Glutamine	0.584	+
Glycine	0.03	+
L-Histidine • HCl • H_2O	0.042	+
L-Isoleucine	0.105	+
L-Leucine	0.105	+
L-Lysine • HCl	0.146	+
L-Methionine	0.03	+
L-Phenylalanine	0.066	+

L-Serine	0.042	+
L-Threonine	0.095	+
L-Tryptophan	0.016	+
L-Tyrosine • 2Na • $2H_2O$	0.10379	+
L-Valine	0.094	+
**Vitamins**		
Choline Chloride	0.004	+
Folic Acid	0.004	-
myo-Inositol	0.0072	+
Niacinamide	0.004	+
D-Pantothenic Acid • ½Ca	0.004	-
Pyridoxine • HCl	0.00404	-
Riboflavin	0.0004	-
Thiamine • HCl	0.004	-
**Other**		
D-Glucose	1	+
Phenol Red • Na	0.0159	-
Pyruvic Acid • Na	0.11	+
